# Bias reduction in representation of histopathology images using deep feature selection

**DOI:** 10.1038/s41598-022-24317-z

**Published:** 2022-11-21

**Authors:** Azam Asilian Bidgoli, Shahryar Rahnamayan, Taher Dehkharghanian, Ali Grami, H.R. Tizhoosh

**Affiliations:** 1 Bharti School of Engineering and Computation, Laurentian University, Sudbury, Canada; 2 NICI Lab, Ontario Tech University, Oshawa, Canada; 3 NICI Lab, Brock University, St. Catharines, Canada; 4 Department of Pathology and Molecular Medicine, McMaster University, Hamilton, Canada; 5 Kimia Lab, University of Waterloo, Waterloo, Canada; 6 Department of Artificial Intelligence and Informatics, Mayo Clinic, Rochester, MN USA

**Keywords:** Cancer, Cancer imaging, Computational biology and bioinformatics, Data acquisition, Data mining, Data processing

## Abstract

Appearing traces of bias in deep networks is a serious reliability issue which can play a significant role in ethics and generalization related concerns. Recent studies report that the deep features extracted from the histopathology images of The Cancer Genome Atlas (TCGA), the largest publicly available archive, are surprisingly able to accurately classify the whole slide images (WSIs) based on their acquisition site while these features are extracted to primarily discriminate cancer types. This is clear evidence that the utilized Deep Neural Networks (DNNs) unexpectedly detect the specific patterns of the source site, i.e, the hospital of origin, rather than histomorphologic patterns, a biased behavior resulting in degraded trust and generalization. This observation motivated us to propose a method to alleviate the destructive impact of hospital bias through a novel feature selection process. To this effect, we have proposed an evolutionary strategy to select a small set of optimal features to not only accurately represent the histological patterns of tissue samples but also to eliminate the features contributing to internal bias toward the institution. The defined objective function for an optimal subset selection of features is to minimize the accuracy of the model to classify the source institutions which is basically defined as a bias indicator. By the conducted experiments, the selected features extracted by the state-of-the-art network trained on TCGA images (i.e., the KimiaNet), considerably decreased the institutional bias, while improving the quality of features to discriminate the cancer types. In addition, the selected features could significantly improve the results of external validation compared to the entire set of features which has been negatively affected by bias. The proposed scheme is a model-independent approach which can be employed when it is possible to define a bias indicator as a participating objective in a feature selection process; even with unknown bias sources.

## Introduction

Under a light microscope with various magnification settings, histopathology is the study of alterations in tissue samples^1^. Histopathology examination is typically required when a definitive diagnosis is needed due to the invasive nature of biopsy techniques^2^. Additionally, pathology is used to diagnose a wide range of inflammatory conditions, infections, and metabolic and autoimmune illnesses^3–6^. A new avenue for the application of computer vision techniques used in computer-aided diagnostic (CAD) systems has been created by the digitization of standard tissue glass slides into Whole Slide Images (WSIs)^7^. One of the prospective uses of computational pathology to aid pathologists is image search^8–10^. It provides search through a WSI database for images with equivalent histomorphological characteristics or a specific region of interest. End-users may discover their first diagnosis, acquire more about the patient’s prognosis, and ultimately arrive at the final diagnostic interpretation when integrating additional data using the information acquired from comparable instances. As a result, content-based image search can aid in the growth of precision medicine.

WSIs are generally gigapixel images (e.g., 100 k $\times$ 100 k pixels) in which a set of much smaller tiles/patches should be extracted to represent each WSI^11^. Then features, as the representatives of histopathological patterns of images should be extracted. Analogous to other computer vision fields, histopathology has also been heavily influenced by the emergence of Convolutional Neural Networks (CNNs) which have become an integral part of digital pathology^12^. Nowadays, many researchers prefer to use pre-trained CNNs for feature extraction^9,10,13^. The quality of features plays a crucial rule in the performance of a digital pathology tasks such as image search and classification. In the medical domain, in order to evaluate the generalization capability of any model, including a trained deep model, “*external validation*” is conducted, in which the model is tested on a set of unseen data. The necessity of external validation in medical image analysis is widely accepted as the accuracy of the model is significantly degraded in many reported cases^14^. One of the possible reasons of this crucial validation process is the potential existence of **bias** in medical images. Avoiding biased data and biased training are crucial challenges in AI^15,16^. Many machine learning methods are unsuccessful on unseen data from similar domains while they achieve highly promising results on training or test sets within the same domain. Additionally, the decisions of an AI algorithm should not reflect discriminatory behavior toward certain groups or populations^17^; various types of bias can negatively affect a data-driven model simultaneously^18^. In a recent study, Howard et al.^19^ reported that the distribution of clinical information in the TCGA data, such as survival and gene expression patterns, remarkably differs among samples provided by various clinics and laboratories. They showed that some models detect source sites instead of predicting prognosis or mutation states; which is neither expected nor desired. Many works have been conducted to eliminate these site-specific signatures to enhance the reliability of histologic image analysis, some through correcting the differences in slide staining between institutions^20^. Some research works tried to utilize the methods proposed by Reinhard et al. and Macenko^21,22^ to decrease color variation across images. Additionally, other works^23,24^ utilized color augmentation, where the color channels are altered at random during training to prevent a model from learning stain characteristics of a specific site. Most research works on stain-normalization and augmentation techniques have focused on model performance in validation sets, rather than elimination of the site-specific signature that may lead to a biased model^25,26^. In addition, bias may exist in any type of medical images. DeGrave et al.^27^ showed that the trained models on radiographic images are more likely to learn medically irrelevant shortcuts, usually attributable to bias in data acquisition, instead of the actual underlying pathology related information. In^28^, the authors revealed that an approach may be subject to bias if the feature extractor is trained on specific institution datasets and potential hidden biases are not accounted for. Factors such as scanner configuration and noise, stain variation and artifacts, and source site patient demographics are more likely potential reasons for the observed biases^28^. The bias detection and recognition would be more challenging when we are faced with multiple types of biases simultaneously. In order to overcome bias in DNNs, some works^29^ tried to find the relationship between the attributes existing in an image to discover those that cannot be well learned by CNN. Authors proposed a method to discover potentially biased representations hidden in a pre-trained CNN. In addition, a representation-based bias may be removed by resampling technique^30^. The proposed method looks for a set of weights at the example level that penalizes samples that are simple for a classifier created using a given feature representation. This is accomplished by learning an independent linear classifier and employing a DNN as a feature extractor for the desired representation. Then, minimizing the difference between this classifier’s loss on the reweighted dataset and the uncertainty of the ground truth class labels is equivalent to the minimizing bias.

In a recent study^28^, the existence of bias in histopathology images of The Cancer Genome Atlas (TCGA)^31^ was investigated. It was established that the deep features extracted from the images are able to accurately distinguish the WSIs based on their acquisition site. Therefore, samples provided by each institution apparently have similar clinical characteristics possibly stemming from various factors, such as digital scanner configuration and noise, tissue stain variation and artifacts, and source site patient demographics. This reveals that DNNs are perhaps unexpectedly detecting the specific irrelevant patterns of source site rather than histomorphologic patterns which results in failure against an external validation. Surprisingly, the bias issue with features is regardless of whether a pre-trained DNN such as DenseNet^32^ is utilized as the feature extractor or a state-of-the-art DNN trained on cancer subtypes such as KimiaNet^33^. In other words, even if a DNN is not specifically trained on histopathology images, it is able to discriminate the sources of the samples. This is likely because the utilized data for extracting the features is still bias-contaminated. Therefore, in addition to the challenge of biased training, apparently there exist some tissue source-site specific patterns in TCGA images (originating from many hospitals) which can be even reflected in features extracted by pre-trained networks. That is possibly related to the non-optimal design of the DNNs structure which leading to extracted non-optimal set of features^34^. Consequently, it seems that DNNs have a tendency to learn irrelevant shortcuts patterns related to data acquisition sites, rather than the actual underlying morphological information^28^.

In order to tackle these bias issues, three categories of solutions can be imagined. Since the bias originates from the institutions that contributed WSIs to the dataset, the issues such as variation in tissue processing, tissue stain protocols, stain quality, color intensity, scanning hardware platforms, and imaging protocols should be considered for filtering the bias; This, however, does not seem to be an easy task to enforce standardization guidines for general bias filtering, at least in foreseeable future. Alternatively, the training methods could probably be modified to avoid incorporating of hospital-identifying factors into images and consequently to improve the generalization of the model^35–37^. However, this cannot be an efficient solution for pre-trained networks or for DNNs which have been already trained on biased data. There are many DNNs that are presently being used for different applications despite of considering the effects of biased data and/or biased training^33,38^.

Motivated by the above considerations, elimination of biased features from the extracted original features can be an efficient and applicable approach as a post-processing solution for downstream tasks. In other words, the features that encompass the knowledge about the source institution can be removed so that the remaining features discriminate the cancer types through relevant information of histopathologic patterns rather than hospital-identifying visual clues. In this direction, we propose a feature selection framework to select a set of optimal features among the output of a feature extractor. An optimal set of features are those that discriminate the cancer types accurately but do not distinguish the images based on their acquisition source, i.e., the hospital. In fact, during the feature selection, we will explicitly enforce removal of the “*bias-contaminated*” features.

In this work, we propose three vital objectives for feature selection as an optimization problem. Similar to all feature selection processes in machine learning, the main goal is to remove redundant and irrelevant features to achieve more accurate image analysis results. Thus, the first objective is defined as the maximization of accuracy of image search. But in addition to the performance of image search, it is desirable to reduce the bias toward source site in optimal feature set. Hence, the accuracy of classification on institute labels is assumed to reflect the bias and consequently is considered as the second objective to be minimized; in fact, the minimization of this objective will manage to remove the bias-contaminated features as much as possible by considering the trade-off between the two objectives.

Finally, shortening the feature vector representing a WSI enables us to sample more tissue patches for each WSI as digital pathology tasks such as image search can be sophisticated with current techniques. Accordingly, A small representative code will be essential for both greatly speeding up image retrieval and utilising less memory. The main contribution of this paper is to design a framework to alleviate the bias as a post-processing technique. To the best of our knowledge, this is the first time that a proposed framework can effectively address the bias issue for pre-trained networks. We tailor the evolutionary algorithms for this purpose. The innovation of this study is not only in designing such a framework but also in proposing three objective functions to formulate the bias in medical images. The discovery of bias formulated by the capability of institution classification using histopathological features and proposing a solution to address it are pivotal for the research and pathologist community. The proposed framework can be utilized as a complementary approach with other bias control methods which work toward cleaning data or reducing bias during training phase; it is model-independent (pre-trained or newly trained ones) and can be utilized when defining a bias indicator is possible with no need to know about the source of the bias.

Briefly, the proposed feature selection can be modeled as a *multi-objective optimization* with three major objectives. We tailored an evolutionary feature selection algorithm to select the best subset of features to increase the generalization performance of a compact representative code for histopathology images by reducing the internal bias and increasing the accuracy of image search simultaneously.

## Results

### A summary of dataset

We utilized TCGA repository which consists of 32,072 WSIs for 32 primary diagnoses. The labeling of these images is at the WSI level (i.e., no pixel-level delineations) and includes information such as ‘morphology’, ‘primary diagnosis’ and ‘tissue or organ of origin’.

Based on a previous study^39,40^, in which a deep network called “*KimiaNet*” has been trained for the purpose of histopathology feature extraction, only the permanent section (i.e., formal fixed, paraffin embedded tissue) diagnostic slides from the TCGA repository were used (i.e., frozen tissues sections were excluded due to their low quality). Cases with no diagnostic, morphological or magnification information reported in addition to the ones scanned at a magnification lower than $20 \times$ were also removed. Another step of the data cleaning was grouping the slides by the combination of their ‘morphology’, ‘primary diagnosis’ and ‘tissue or organ of origin’ and removing the ones with less than 20 instances, so each group has at least 2 slides available for the test set. This resulted in removing 2 of the 32 primary diagnoses, which are UCEC (Uterine Corpus Endometrial Carcinoma) and DLBCL (Diffuse Large B-cell Lymphoma).

According to^41^, images of TCGA are categorized into 12 tumor sites. These categories include: endocrine, hematopoietic, pulmonary, breast, brain, gastrointestinal tract, melanocytes, gynecological, prostate/testis, liver/pancreaticobiliary, urinary tract, and mesenchymal. Each tumor site except mesenchymal, hematopoietic, and breast, consists of more than one primary diagnosis which enables classification task. Table 1 represents the number of samples and defined identity (ID) for each primary diagnosis.

**Table 1:** The information of TCGA dataset.

Tumor type	Subtype	ID	#Test samples	#Site sources
Brain	Brain lower grade glioma	LGG	35	19
	Glioblastoma multiforme	GBM	39	
Endocrine	Adrenocortical carcinoma	ACC	6	22
	Pheochromocytoma and paraganglioma	PCPG	15	
	Thyroid carcinoma	THCA	51	
Gastrointestinal	Colon adenocarcinoma	COAD	33	22
	Rectum adenocarcinoma	READ	11	
	Esophageal carcinoma	ESCA	14	
	Stomach adenocarcinoma	STAD	30	
Gynecological	Cervical squamous cell carcinoma and endocervical adenocarcinoma	CESC	17	11
	Ovarian serous cystadenocarcinoma	OV	10	
	Uterine carcinosarcoma	UCS	3	
Liver	Cholangiocarcinoma	CHOL	4	17
	Liver hepatocellular carcinoma	LIHC	35	
	Pancreatic adenocarcinoma	PAAD	12	
Mesenchymal	Uveal melanoma	UVM	4	13
	Skin cutaneous melanoma	SKCM	24	
Prostate/testis	Prostate adenocarcinoma	PRAD	40	21
	Testicular germ cell tumors	TGCT	13	
Pulmonary	Lung adenocarcinoma	LUAD	43	25
	Lung squamous cell carcinoma	LUSC	38	
	Mesothelioma	MESO	5	
Urinary tract	Bladder urothelial carcinoma	BLCA	34	29
	Kidney chromophobe	KICH	11	
	Kidney renal clear cell carcinoma	KIRC	50	
	Kidney renal papillary cell carcinoma	KIRP	28	

The tumor type categorization, tumor subtypes (primary diagnosis), short ID of each primary diagnosis, the number of test samples for each tumor, and the number of site sources for test samples are reported.

### Deep features and WSI indexing

For the specific purposes of this work, we extracted two sets of features from the patches of the whole dataset, using “KimiaNet”^40^ as a network specifically trained on histopathology images and “DenseNet-121”^32^ as a pre-trained network. DenseNet-121 is a compact architecture with almost 7M parameters. The network is trained by 1.2 million natural images from ImageNet^42^. The architecture of KimiaNet is the same as DenseNet-121 but re-trained from scratch on TCGA histopathology images. The features are extracted from the last pooling layer.

On high-cellularity patches of TCGA images, KimiaNet is trained using soft labels. For the training, validation, and test datasets, respectively, the authors split the patches into 7126, 741, and 744 diagnostic WSIs during pre-processing processes. In order to extract the features based on the 30 primary diagnoses, the network is trained using a set of patches. As a consequence, a WSI is characterized by 135 feature vectors, each in size of 1024. The mean of feature vectors (MFV) over all patches is utilised as a useful representation in order to reduce the amount of data needed for WSI representation. In this manner, a WSI is represented by the mean of feature vectors that only have 1024 values before to the feature selection. Because KimiaNet was trained on *high cellularity* patches, which impose homogeneity, MFV is relevant in our application.

In order to distinguish the primary diagnosis throughout the full dataset, as was already indicated, feature vectors are extracted (i.e., discrimination of 32 cancer types). However, the primary objective of an image retrieval system is to identify the images from a particular category of tumour type that are most similar to a given query image.

### Bias reduction in representation of WSIs

**Figure 1:**
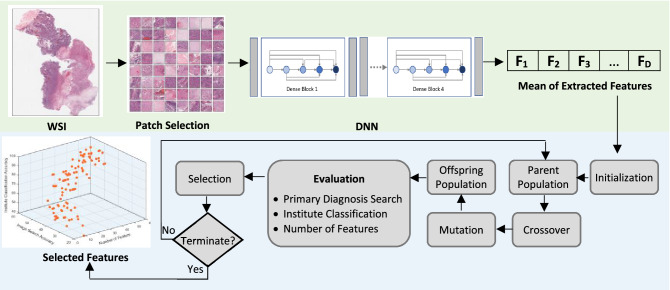
The overall process of the removing the features with carrying highest amount of bias. The green block represents the steps of data patch selection and feature extraction whereas the optimization process is presented in the blue block. The final output is a set of non-dominated solutions (i.e., feature subsets). *D* reflects the number of extracted features.

There exists significant bias toward the source institutions in extracted features by deep networks. This implies that DNNs may have learned to distinguish image source institutions as a form of biased shortcut to classify cancer types. If we assume that the features comprise of both relevant histologicall information and irrelevant non-morphologic tissue source site patterns, then, an optimal feature selection may be a logical approach to eliminate the biased features. Therefore, the proposed method aims to decrease the undesirable bias by selecting the optimal features set which distinguish the cancer types independently from institutes. Accordingly, our solution builds upon the concept of a post-processing phase of learning regardless of the type of data-driven feature extractor. Figure 1 illustrates the overall structure of the process proposed to generate a set of features to alleviate bias effects in histopathology image search.

As mentioned previously, the features are extracted from the images of TCGA repository which includes 12 tumor type categories each of which encompasses several primary diagnoses (tumor subtypes) resulting in 32 primary diagnoses in overall. For each tumor type, an optimal set of relevant features are selected among all extracted features to accurately classify the primary diagnosis related to corresponding tumor type. By this way, the multi-objective optimizer tries to eliminate all redundant, irrelevant, and biased features which is tailored for each tumor type accordingly.

In the light of the aforementioned points, feature selection is defined as a multi-objective optimization problem which can be solved by an evolutionary algorithm. Evolutionary algorithms have strong global optimization capabilities and can consider the combination of features in terms of multiple objectives^43^. An optimization model is made up of four main components: the objective functions that we intend to minimize or maximize, the problem encoding, the constraints of the problem (if any), and the optimization algorithm.

The fallowing three objectives are defined to select the optimal features in order to decrease the bias resulting from acquisition sites:

1. **Maximization of the image search quality. ** Mainly, the evolutionary feature vector is expected to increase the efficiency of image retrieval. In order to evaluate the quality of selected features, the most similar slides to a query WSI are retrieved. By removing redundant and irrelevant features, the best match of query image can be found. Evaluation of the image search is accomplished by calculating the F1-score of k-Nearest Neighbor (KNN) method on primary diagnosis labels^33^. As mentioned previously, one of the prospective uses of computational pathology is image search which provides case matching through a WSI database for images with equivalent histomorphological characteristics or a specific region-of-interest. We utilized the kNN technique to determine the top matched (i.e., the most similar) features when we searched through the features. However, to evaluate the performance of search, we have used KNN as a search-based classifier to quantify the efficiency of features. The KNN is one of the commonly used methods in this field. Therefore, the F1-score of image search is considered as the first objective, $f_1$, for a set of selected features, *S*. F1-score is a harmonic mean of precision and recall measures defined as the follows^44^.
  1 $$\begin{aligned} f_1(S)=F1\text{- }score = 2\times \frac{\text {Precision}\times \text {Recall}}{\text {Precision}+ \text {Recall}}, \end{aligned}$$
  where *Precision* can be calculated as
  2 $$\begin{aligned} Precision = \frac{\text {True Positive}}{\text {True Positive} + \text {False Positive}}. \end{aligned}$$
  Similarly, *Recall* can be given as
  3 $$\begin{aligned} Recall = \frac{\text {True Positive}}{\text {True Positive} + \text {False Negative}} \end{aligned}$$
2. **Minimization of the number of required features. ** When building a classification model on high-dimensional data, the “curse of dimensionality” is a crucial problem, according to^45^. A shorter code can reduce the complexity of memory needs while simultaneously speeding up retrieval in datasets with many gigapixel pictures. Even when there are less characteristics, there may still be insufficiently relevant information for the learning system to accurately categorize WSIs^46^. As a result, it is thought that the size of the feature vector and classification accuracy are two conflicting objectives. In the optimization phase, a string with a length equal to the number of features is used to represent each individual for feature selection. A feature’s status is represented by a cell in the vector, where a value of ‘0’ denotes a feature’s rejection and a value of ‘1’ denotes a feature’s selection.Therefore, the number of selected features is equivalent to the total number of ‘1’s in the vector. Hence, given a set of all features represented by a binary vector, $m_1,m_2,\ldots ,m_D$, the ratio of selected features, *S*, as the second objective, $f_2$, is calculated as follows:
  4 $$\begin{aligned} f_2(S)=\frac{1}{D}\sum \limits _{i=1}^{D}m_i, \end{aligned}$$
  where *D* is the total number of features.
3. **Minimization of the bias of image acquisition source.** On the flip side, the bias toward the acquisition source can be defined by the accuracy of institute classification. Therefore, the third objective of feature selection can be reasonably defined as the minimization of institute classification accuracy over the selected features. Ideally, the extracted features should not be the representatives of their source institute. Hence, accurate classification of acquisition sites using the features reveals the existence of bias and may inevitably lead to considerable drop in accuracy for processing of external data (i.e., images from unseen hospitals). In order to avoid the bias, the optimization algorithm explores the search space of features to find the optimal set of features with minimum accuracy of institute classification. Similar to what we done for image search accuracy, the quality of selected features for constructing a model to accurately classify the institutions is assessed. To this effect, each image is labeled with the cooperated institution instead of primary diagnosis. The best of features should not represent the information related to institutions, hence, we select a set of features with minimum accuracy of institution classification. Hence, the third objective, $f_3$, for a set of selected features, *S*, is calculated as follows:
  5 $$\begin{aligned} f_3(S)= \text {Accuracy} = \frac{\text {True Negative} + \text {True Positive}}{\text {total number of samples}}. \end{aligned}$$

### Results on TCGA dateset

In this section, we explain the conducted experiments in order to assess the proposed feature selection for gigapixel pathology images to decrease the bias. The series of experiments investigate the effectiveness of selected features in terms of the value of objectives. The main goal of experiments is to observe whether the optimizer is able to select an optimal set of features with minimum bias toward image acquisition source.

In this study, 12 independent tumor-based optimization problems are defined to decrease the number of features and to select the optimal subset specialized for each tumor type category by decreasing the bias, increasing the image search accuracy, and decreasing the number of selected features, simultaneously.

Following some preliminary trials, the population size and the number of fitness calls were set at 50 and 512,000, respectively. The validation set images are searched among the training set using the *k*-nearest neighbour technique^47^. In order to assess the potential solutions during the optimization process. To get the average F1-score across all primary diagnoses, three images that are the most close in terms of Euclidean distance are obtained. The second goal is to determine how many features were chosen. In order to classify the images based on their institutes of origin, we need to employ one of the state-of-the-art classification algorithms. To this effect, a one-versus-all Support Vector Machine (SVM) with linear kernel is trained on the training images. The SVM is a machine learning method that has become exceedingly popular for its simplicity, flexibility, and effectiveness for addressing a range of classification problems. SVMs stand out for their ability to provide balanced predicted performance, even in research with potentially small sample numbers^48^.

The corresponding subset of features is selected in training set and then the accuracy is obtained on validation set as the third objective. Due to the stochasticity of evolutionary algorithms, according to^49^, the experiments are conducted as 31 independent runs resulting in 31 Pareto-fronts. The final feature subsets on Pareto-front are evaluated on the test set. Finally, the Wilcoxon statistical test^50^ is conducted to analyze the significance of the generated results.

**Figure 2:**
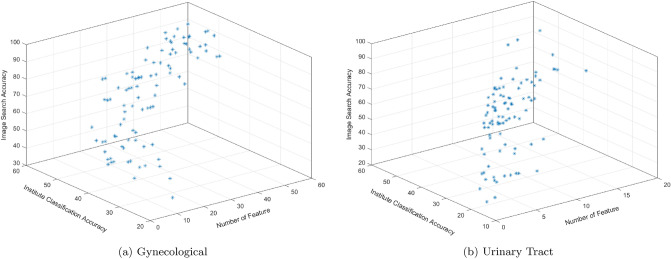
Non-dominated solutions for two samples of primary sites, Gynecological and Urinary Tract. Trade-off solutions are presented in terms of the values of three objectives. The minimum number of features, maximum image search accuracy, and minimum classification accuracy per institution are desired values for a resultant feature subset.

Figure 2 illustrates the resultant Pareto-fronts (i.e., non-dominated solutions) for two sample tumor sites. There are a set of trade-off solutions at the end of the optimization process. For those subsets with more features, the image search accuracy is higher and also the hospitals/institutions are classified less accurately. However, the range of resultant objective values can be various for different sites. As it can be seen, the optimizer selected the subsets with maximum 60 features to distinguish primary diagnoses of gynecological whereas the maximum number of features for urinary tract is 20.

In addition, Table 2 represents the results of evolutionary features in terms of F1-score of image search and classification accuracy of hospitals/institutions. From all non-dominated candidate solutions, we picked the feature subset with maximum F1-score on tumor type search. As previously mentioned, the total number of features is 1024 whereas the evolutionary algorithm selected 13 and 15 features on average among the KimiaNet and DenseNet features, respectively. This considerable drop may be attributed to two different factors. First, because the network is generally trained to identify all sorts of cancers (i.e., 32 classes), only a very limited fraction of features may be chosen for a given tumour category consisting up to four primary diagnosis. Second, it’s possible that the trained DNNs’ topology is not optimal, which might cause it to extract a lot of useless information during training. The proposed approach therefore selects a subset of evolutionary features for each tumour category in order to address the sub-optimality of retrieved information. This fraction can then more successfully and effectively carry out downstream tasks like retrieval and classification. From the table, extracted KimiaNet features (i.e., 1024) are able to classify the acquisition institutions with higher accuracy, i.e., 74.91%; however, the model is inherently trained for cancer subtype classification. This reveals the high bias of contributing institutions in the learning process of KimiaNet. The evolutionary algorithm decreased the number of required features for image search to 13 features on average whereas the F1-score of tumor search is increased to 87.19% as well. Interestingly, the accuracy of institute classification using the selected features is significantly reduced which results in 34.87% accuracy (i.e, 40% reduction). It is observed that the proposed evolutionary feature selection can eliminate the features leading to high bias of institute classification and selects those which augment the tumor search accuracy.

From Table  2, different accuracy values can be seen for various classes. This comes from the nature of the cancer types; for some classes such as Brain, the discrimination between the pathological patterns of two cancer subtypes (i.e., LGG and GBM) is sufficiently high and consequently, the differentiation is not difficult for classification algorithm. While for some organs, such as Gastrointestinal, there are some overlapping patterns between the samples of READ and COAD classes which causes the difficulty for algorithms to differentiate them.

Similar results are obtained using DenseNet features. Although DenseNet is a pre-trained network with no learning process on histopathology images, the classification accuracy of institutions is still high (i.e., 68.97%); just 6% less than the KimiaNet case which was trained with the biased data. This suggests that not only the learning process of deep networks can be biased by information of acquisition sites but also the TCGA images contain the clues of source institutions which are picked up by network during the feature extraction. Surprisingly, the accuracy of institution classification indicating the bias is even higher than the average F1-score of tumor subtype search. As it can be seen from the Table 2, selected evolutionary features could improve the image search based on the cancer type to 66.68% whereas the accuracy of institute classification is decreased to 32.29% (i.e., 36% reduction) as a result of conducted features selection.

According to the experimental results, the existence of bias toward the information of tumor site sources is not deniable. However, the elimination of such bias in extracted features can be accomplished as a post-processing phase applicable on any data-driven features extraction method. The proposed evolutionary algorithm selects those features that contain less discriminative patterns of source site institution and instead keep the expressive information to distinguish the cancer subtypes. By decreasing the undesirable correlation between the cancer type information and their source sites, we expect the selected features to lead to more accurate external validation, i.e., higher generalization. Therefore, even if models learn transiently useful but histologically irrelevant information from the images, the proposed feature selection can be effective to eliminate those features irrelevant to primary diagnosis patterns and consequently to prevent their negative impacts on WSI representation. The reported results clearly demonstrate that the idea of embedding bias reduction objective in the multi-objective feature selection process can promisingly reduce the bias up to 40% and 36% over the considered two state-of-the-art DNNs as the case studies.

**Table 2:** The comparison between the results of all 1024 features extracted by KimiaNet and DenseNet and evolutionary features.

Tumor type	Subtype	#WSIs	KimiaNet					DenseNet				
			All features (1024)			Feature selection		All features (1024)			Feature selection	
			Institute class	Tumor search	#F	Institute class	Tumor search	Institute class	Tumor search	#F	Institute class	Tumor search
Brain	LGG	35	84.5	84.51	10	**34.98**	**86.49**	77.87	77.78	14	38.14	**83.33**
	GBM	39		85.71			**86.49**		78.95			**84.21**
Endocrine	ACC	6	66.53	54.55	14	**31.16**	**100**	56.32	18.18	10	39.13	**46.15**
	PCPG	15		83.87			**100**		**57.14**			51.85
	THCA	51		**100**			**100**		89.52			**90.38**
Gastrointestinal	COAD	33	76.5	**76.71**	3	**32.25**	75.36	81.7	53.66	7	30.56	**55**
	READ	11		**42.11**			22.22		22.22			**50**
	ESCA	14		**84.62**			46.15		20			**38.10**
	STAD	30		**79.31**			76.19		**64.29**			47.27
Gynecologic	CESC	17	58.42	**97.14**	40	**43.66**	**97.14**	54.55	83.33	13	27.97	**87.18**
	OV	10		**94.74**			**94.74**		70			**75**
	UCS	3		**100**			**100**		0			**80**
Liver	CHOL	4	83.6	50	2	**48.87**	**85.71**	73.5	0	37	49.88	**36.36**
	LIHC	35		95.77			94.59		85.29			**89.55**
	PAAD	12		73.68			**85.71**		56			**75**
Mesenchymal	UVM	4	74.67	66.67	19	**30.04**	**85.71**	74.1	40	3	30.72	**66.67**
	SKCM	24		96			**97.96**		94.12			**96**
Prostrate	PRAD	40	73.72	**100**	17	**48.48**	**100**	75.81	**96.39**	3	34.69	95.12
	TGCT	13		**100**			**100**		**86.96**			83.33
Pulmonary	LUAD	43	81.48	**81.58**	3	**17.92**	**81.58**	63.86	**67.47**	17	7.13	50.63
	LUSC	38		**86.36**			83.72		**66.67**			53.49
	MESO	5		75			100		0			**57.14**
Urinary tract	BLCA	34	74.81	94.29	10	**26.45**	**97.14**	63	**82.67**	30	32.37	82.05
	KICH	11		90			**90.91**		**38.10**			33.33
	KIRC	50		**95.05**			94		**78.79**			70.83
	KIRP	28		**87.27**			85.19		**70.59**			55.56
Avg.		$\approx$23	74.91	83.65	$\approx$13	**34.87**	**87.19**	68.97	57.62	$\approx$14	**32.29**	**66.68**

Significant values are in [bold].

The F1-score of image search by 3-nearest neighbors, the classification accuracy of institutions, and the number of selected features are reported.

**Figure 3:**
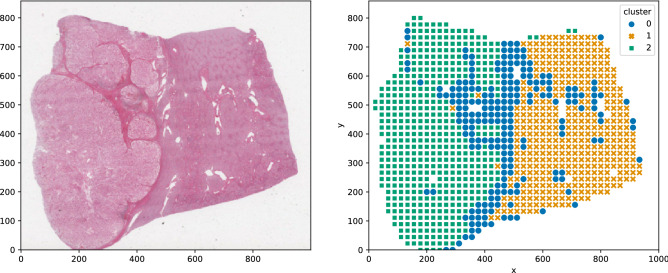
K-means clustering was applied on the optimized subset of features to cluster tissue patches of a renal clear cell carcinoma into 3 classes. It shows that each cluster correlates to a different tissue type. Clusters 1, 2, and 3 correspond to fibrous tissue, normal renal tissue, and malignant regions, respectively.

In order to transparently show the performance of selected features, it is essential to evaluate the capability of the optimized features to distinguish different tissue types of WSIs. K-means clustering was applied to cluster tissue patches of a renal clear cell carcinoma sample, using only the optimized subset of features to represent each tissue patch. The k was arbitrarily set to 3. The result is shown in Fig.  3. One can see that each cluster corresponds to a specific type of tissue. This visualization suggests that the optimized subset of features is both accurate for cancer type classification and retains its capacity to distinguish tissue types within a specimen.

### External validation

As mentioned previously, the destructive effect of existing bias in a dataset utilized for training a DNN is mostly on external data, i.e., the unseen samples from an institution that is not participated in training or test. We showed that we could reduce the bias on TCGA data by considering it as an objective function in our optimization. However, the institutions of images in test data had also participated in training and optimization phases. In order to investigate the benefits of the proposed method on generalization for the search, it is required to conduct external validation. Hence, the performance of the proposed method is validated using a set of external Kidney images which have not participated in neither training nor optimization process. The dataset consists of 142 samples of Kidney cancer subtypes from the Ohio State University. There exist 50 samples from KIRC subtype, 45 from KICH, and 50 from KIRP according to subtypes presented in Table 1. Similar to what we did for TCGA images, for each WSI, a set of patches are extracted. The KimiaNet is utilized to generate the 1024 features for each patch. The mean of all feature vectors is the representative of each WSI. The comparison is required between the accuracy of search of unseen samples using all extracted features (i.e., 1024) and selected features using the proposed method. The optimal features are output of the previous experiments (i.e., on TCGA images). Then each image of external data is considered as a query to search among the rest of images. The average F1-score resulting the search of all images is considered as the evaluation measure. Table 3 presents the output of this comparison. As it can be seen, the F1-score values for searching the images among the external data are 86.05, 75, and 78.43 for KICH, KIRC, and KIRP, respectively. Whereas the selected features using the proposed method could achieve 86.32, 84.78, and 86.6 for KICH, KIRC, and KIRP, respectively, which shows 6% improvement in average. As indicated in Table 2, the average value of F1-score on test data using all features was 90.77 while this number decreases to 79.83 on data from an external institution. This reveals that although the test data is necessary for assessing the accuracy of AI models, it is not sufficient to validate their performance against the bias. Accordingly, external validation becomes essential to show how the model performs on data from an external resource. Feature selection based on the minimization of the accuracy of institution classification alleviates the impact of bias as the F1-score drops only 4.87% on the search of external data.

**Table 3:** A comparison between the results of external validation using all features and the selected features.

	KICH	KIRC	KIRP	Avg.
#WSIs	45	50	50	48.33
All features (1024)	86.05	75	78.43	79.83
Selected features	86.32	84.78	86.60	85.90

## Discussion

In this work, we proposed a novel feature selection framework to reduce the effect of the biased features of DNNs when operating on histopathology images. The proposed framework is applied to the extracted features from the images as the output of the DNNs after they have been trained. Therefore, the presented bias reduction strategy is independent of the utilized dataset and the DNN itself since it operates as a post-processing stage of a learning framework. Feature selection aims to eliminate the irrelevant and redundant features that degrade the image analysis performance and remove the bias-contaminated features carrying the image acquisition site-specific patterns unintentionally present in digital slides. To this effect, three objectives, including maximization of image search accuracy, minimization of institution classification accuracy, i.e., defined as the bias indicator, and minimization of the number of features for compactness, are considered. Two sets of experiments were conducted on features extracted using a pre-trained network and a DNN trained on histopathology images. The experimental results on NIH’s TCGA repository showed that the optimal features result in significantly lower classification accuracy of institution labels, alleviating the feature vector’s internal bias. However, the resultant representation code is highly compact and accurate for primary diagnosis discrimination. As one of the valuable experiments, we conducted external validation to clearly show the performance of selected optimal features on the unseen data from an institute that is not contributed to any part of the development of the method. This experiment clearly showed that the selected optimal features take advantage of the bias reduction and can search the images more accurately than all features. In conclusion, we have demonstrated in this paper that the proposed evolutionary framework can be beneficial in improving the generalization capability of feature extractors by alleviating the degrading impact of biased data on generalization and medical image analysis. This work demonstrated the potential for new complementary post-processing to overcome bias in deep learning.

The only assumption we make is that we can select the optimal features to minimize the accuracy of institute/source classification. The group structure of genetic data might be useful in finding the relation between the genetic information and cancerous patterns but most likely after removing the bias. In this study, we just tried to select the best features with focus on alleviating the bias. The proposed method has formulated three objective functions which can be effective in decreasing bias and increasing the cancer type discrimination at the same time. This framework is designed based on the limited information that we could obtain to interpret the existence of bias in TCGA dataset. Definitely, as a future perspective and with more discovery on the source of bias, the complementary information^51,52^ will be more beneficial to address this issue. Clearly, in this direction, a reduced number of features will make the attempt of explainability more easier.

## Methods

Proposed feature selection framework is designed based on a multi-objective optimization process. Accordingly, we need to define the objective functions based on which features are selected. The process of evolutionary feature selection is explained in the following subsections. By this type of process, a set of random combinations are initialized as a population and as the algorithm iterates, optimizer improves the individuals to find an optimal set of features considering the objective functions. The improvement of individuals (i.e., feature subsets) can be done using a couple of generative operators to create new combinations.

### Multi-objective optimization (MOO)

MOO targets handling two or more conflicting objectives. The use of evolutionary algorithms has been very promising for solving such problems^53^. The population-based nature of these algorithms results in generating a set of candidate solutions at each run of the algorithm. Collaboration of individuals to make an optimal “Pareto-front”^49^ is the core reason for the success of population-based algorithms. The mathematical definition of multi-objective optimization problem can be defined as^54^

6 $$\begin{aligned} \begin{aligned}{}&Min/Max\ F(\pmb x)=[f_{1}(\pmb x),f_{2}(\pmb x),\ldots ,f_{M}(\pmb x)], \end{aligned} \end{aligned}$$

subject to

7 $$\begin{aligned} \begin{aligned}{}&L_{i}\le x_{i}\le U_{i},&i=1,2,\dots ,d,\\&g_i(\pmb x) \le 0 \quad&j=1,2,\dots ,J,\\&h_k(\pmb x)=0 \quad&k=1,2,\dots ,K, \end{aligned} \end{aligned}$$

where *M* is the number of objectives, *d* is the number of decision variables (i.e., dimension), and the value of each variable, $\pmb x_{i}$, is in interval $[L_{i},U_{i}]$ (i.e., box-constraints). $f_{i}$ represents the objective function, which should be minimized/maximized.

Due to the conflicting of objective functions in a multi-objective optimization problems, the definition of the optimality is not as simple as the single-objective case. Therefore, it is required to find a trade-off among objective functions. One of the commonly used concepts for comparing candidate solutions in such problems is the concept of dominance which is defined as follows: If $\pmb x=(x_{1},x_{2},\ldots ,x_{d})$ and $\pmb x'=(x'_{1},x'_{2},\ldots ,x'_{d})$ are two vectors in the problem search space, $\pmb x$ dominates $\acute{\pmb x}$ ($\pmb x\succ \acute{\pmb x}$) if and only if

8 $$\begin{aligned} \begin{aligned}{}&\forall i\in {\{1,2,\dots ,M\}}, f_i(\pmb x)\le f_i(\pmb x') \wedge \\&\exists j \in {\{1,2,\dots ,M\}}: f_j(\pmb x)<f_j(\pmb x') \end{aligned} \end{aligned}$$

This concept defines the optimality of a solution in a multi-objective space. Candidate solution $\pmb x$ is better than $\pmb x'$ if it is not worse than $\pmb x'$ in any of the objectives and at least it has a better value in one of the objectives. All solutions that are not dominated using any other solution called non-dominated solutions; they create the first Pareto-front (Pareto-front) set. Multi-objective algorithms attempt to find these solutions by utilizing generating strategies/operators and selection schemes. The non-dominated sorting (NDS) algorithm^55^ is one of the popular selection strategies which works based on the dominance concept. It ranks the solutions of the population in different ranks of optimality, called Pareto. The algorithm starts with determining all non-dominated solutions in the first rank. In order to identify the second rank of individuals, the non-dominated vectors are removed from the set to process the remaining candidate solutions on the same way. Non-dominated solutions of this step make the second level of individuals (second Pareto). Thereafter, the second ranked individuals will be removed to identify the third Pareto. This process will continue until the all individuals are grouped into different ranks of Pareto.

### Proposed process of evolutionary feature selection

An optimization model is made up of four main components: the objective functions that we intend to minimize or maximize, the problem encoding, the constraints of the problem (if any), and the optimization algorithm. In the following, each component for a multi-objective feature selection problem is defined. In addition to the defined objective functions in the previous section, the optimization requires an encoding scheme to be considered as the representation of variables of the problem. The encoding can represent each individual in the population, demonstrating a set of features. To this end, a binary vector in size of all features indicates the status of a feature, where 0 reflects the absence of a feature and 1 is indicative of feature’s presence. Thus, the optimizer explores the search space to find the best value (i.e., 0/1) for each variable. The overall structure of the proposed framework is illustrated in Fig. 1. The process starts with the steps of pre-processing and features extraction using a DNN, then the optimization is conducted. The output of the optimization process is a set of trade-off solutions; each of trade-off solutions can be selected by an expert decision maker.

We now highlight the steps of the algorithm; similar to all population-based evolutionary algorithms, as it is presented in Fig. 1, the optimization process starts with a initial population of random binary individuals. Each individual is indicator of a set of selected features as a candidate solution. The evolutionary algorithms try to find the optimal solutions by a repetitive process. The algorithm evaluates the initial population based on three defined objectives. By considering these objectives, the optimizer removes the redundant and irrelevant features gradually and keeps the best combination of features in the population. To calculate the accuracy of image search using each subset of features, the training and validation sets of WSIs are adjusted accordingly and the label of each image in validation set is predicted. As previously mentioned, each WSI has “two labels” including (1) the cancer type, and (2) source institute. Thus, the accuracy of prediction is separately calculated based on both labels to declare two objectives.

The next step of the optimization is generating the new individuals in order to explore the search space. One-point crossover is utilized to combine the two individuals in the population to create a new candidate solution. By this operator, a random combination point is selected for both parents individuals. The variables after the combination point are swapped with each other. Two new individuals (i.e., offsprings) are generated. Then bit-wise mutation is utilized in which a number of bits flip at random positions. The new individuals compete with the old population while the best solutions (i.e., non-dominated solutions) are selected for the next generation. We applied the well-known many-objective optimization algorithm to find a subset of features to alleviate the impact of bias. Among these algorithms, the NSGA-III is one of the state-of-the-art algorithms that has been proposed to address the shortcomings of its predecessor NSGA-II. In^49^, the performance of this algorithm has been compared with several algorithms to demonstrate the effect of adding the reference points to improve the distribution and quality of the resulting Pareto front.

The NSGA-III selection strategy chooses the top ranked feature subsets based on the NDS algorithm and the reference lines distributed uniformly in the search space to provide the well-spread out candidate solutions.Those subsets of features (i.e., individuals) with maximum accuracy of primary diagnosis classification and minimum source institution classification (i.e., bias) are selected among the population. In fact, the elite individuals which are the best subsets of features are selected to make the next generation. The evolutionary process leads to optimize the defined objective function for the goal of feature selection. At the end of process, a set of trade-off solutions are obtained. A decision maker can pick one of the solutions according to the desired objective or criterion.

## Data Availability

The TCGA dataset used and analysed during the current study is publicly available in “https://portal.gdc.cancer.gov.”
